# Hobby Engagement and Risk of Disabling Dementia

**DOI:** 10.2188/jea.JE20210489

**Published:** 2023-09-05

**Authors:** Takumi Matsumura, Isao Muraki, Ai Ikeda, Kazumasa Yamagishi, Kokoro Shirai, Nobufumi Yasuda, Norie Sawada, Manami Inoue, Hiroyasu Iso, Eric J Brunner, Shoichiro Tsugane

**Affiliations:** 1 Public Health, Department of Social Medicine, Osaka University Graduate School of Medicine, Osaka, Japan; 2 Department of Public Health, Juntendo University Graduate School of Medicine, Tokyo, Japan; 3 Department of Public Health Medicine, Faculty of Medicine, and Health Services Research and Development Center, University of Tsukuba, Ibaraki, Japan; 4 Department of Public Health, Kochi University Medical School, Kochi, Japan; 5 Epidemiology and Prevention Group, Institute for Cancer Control, National Cancer Center, Tokyo, Japan; 6 Institute of Epidemiology and Health Care, University College London, London, UK

**Keywords:** hobby engagement, disabling dementia, follow-up study, epidemiology

## Abstract

**Background:**

The association between hobby engagement and risk of dementia reported from a short-term follow-up study for individuals aged ≥65 years may be susceptible to reverse causation. We examined the association between hobby engagement in age of 40–69 years and risk of dementia in a long-term follow-up study among Japanese, including individuals in mid-life, when the majority of individuals have normal cognitive function.

**Methods:**

A total of 22,377 individuals aged 40–69 years completed a self-administered questionnaire in 1993–1994. The participants answered whether they had hobbies according to the three following responses: having no hobbies, having a hobby, and having many hobbies. Follow-up for incident disabling dementia was conducted with long-term care insurance data from 2006 to 2016.

**Results:**

During a median of 11.0 years of follow-up, 3,095 participants developed disabling dementia. Adjusting for the demographic, behavioral, and psychosocial factors, the multivariable hazard ratios of incident disabling dementia compared with “having no hobbies” were 0.82 (95% confidence interval [CI], 0.75–0.89) for “having a hobby” and 0.78 (95% CI, 0.67–0.91) for “having many hobbies”. The inverse association was similarly observed in both middle (40–64 years) and older ages (65–69 years). For disabling dementia subtypes, hobby engagement was inversely associated with the risk of dementia without a history of stroke (probably non-vascular type dementia), but not with that of post-stroke dementia (probably vascular type dementia).

**Conclusion:**

Hobby engagement in both mid-life and late life was associated with a lower risk of disabling dementia without a history of stroke.

## INTRODUCTION

A hobby is defined as an enjoyable leisure activity in which individuals actively engage at their own initiative in their free time from work or other responsibilities.^1^ A hobby includes cognitive and physical leisure activities, and engagement in these activities was associated with a lower risk of dementia.^2^^–^^13^ Enjoyable leisure activities were also correlated with a higher level of life engagement (purpose in life),^1^ probably lowering dementia risk.^14^ However, evidence on the relationship between hobby engagement and the risk of dementia is limited.^6^^,^^8^ Furthermore, previous studies examining the association between hobby engagement and risk of dementia recruited individuals aged ≥65 years and followed up their participants for less than 7 years at mean,^6^^,^^8^ probably resulting in reverse causation because a cognitive decline may restrict engagement in hobbies. To minimize the impact of reverse causation, it is necessary to evaluate hobby engagement in mid-life, when the majority of individuals have normal cognitive function, and/or conduct a long-term follow-up survey.

Therefore, we aimed to examine the association between hobby engagement in age of 40–69 years and the long-term risk of dementia in the Japan Public Health Center-based prospective (JPHC) Study Cohort II. We hypothesized that having hobbies was associated with a lower risk of dementia.

## METHODS

### Study population

The JPHC Study Cohort II was a population-based cohort study that started in 1993, enrolling residents aged 40–69 years from six PHC areas: Nagaoka, Niigata Prefecture; Mito, Ibaraki Prefecture; Suita, Osaka Prefecture; Chuo-higashi, Kochi Prefecture; Kamigoto, Nagasaki Prefecture; and Miyako, Okinawa Prefecture. A detailed description of the JPHC study protocol has been published elsewhere.^15^ Briefly, the participants were provided with a self-administered questionnaire about demographic characteristics, medical history, physical activity, smoking, drinking, socioeconomic status, and dietary habits in 1993–1994, and thereafter, were followed up on their health outcomes of disabling dementia ascertained from the long-term care insurance (LTCI) data. Under the Japanese LTCI system starting in 2000, the long-term care service was provided with co-payment to persons aged ≥65 years who need support or care for their activities of daily living (ADL), and those aged 40–64 years who need care due to having specified aging-related diseases, including dementia.^16^^,^^17^ In four towns (Iwase district in Sakuragawa city and Tomobe district in Kasama city in Ibaraki Prefecture; and Kagami and Noichi districts in Konan city in Kochi Prefecture), the LTCI data were available from January 1, 2006, to December 31, 2016, through the approval of local municipalities for the provision of the LTCI data. A total of 13,251 men and 13,976 women were included in the study.

**Figure 1.**
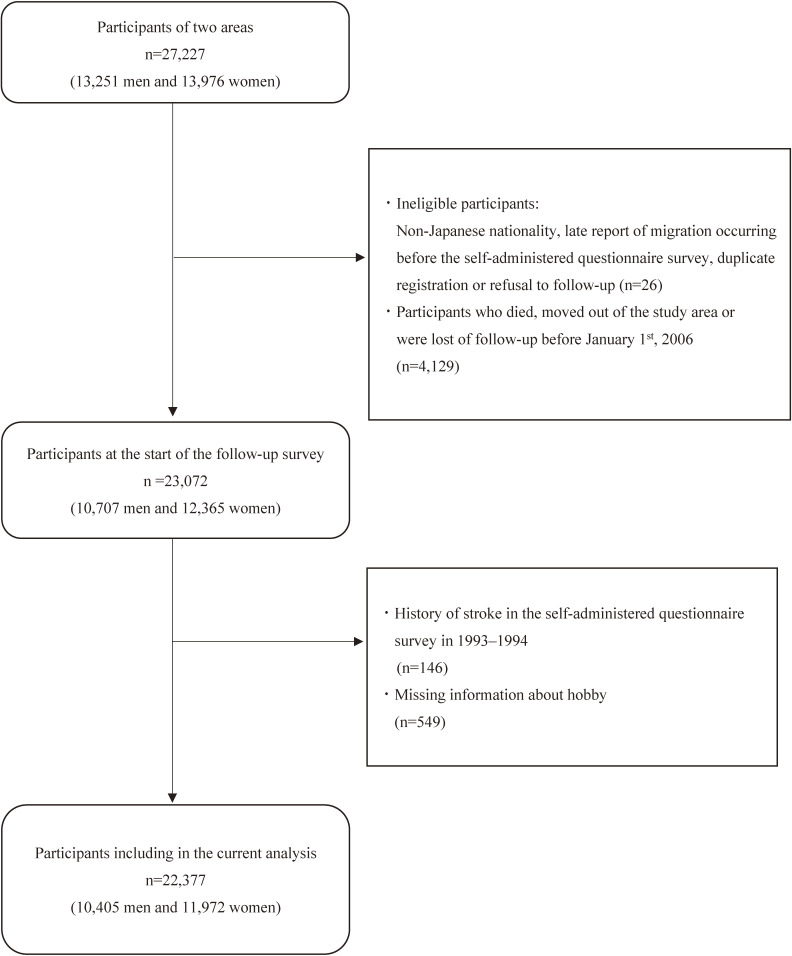
Flowchart of study participants

In the current study, we excluded participants with ineligible criteria: non-Japanese nationality, late report of migration occurring before the self-administered questionnaire survey, duplicate registration, or refusal to follow-up survey (*n* = 26); and those who died, moved out of the study area, or were lost to follow-up before January 1, 2006 (*n* = 4,129). Participants who had a history of stroke in the self-administered questionnaire survey in 1993–1994 (*n* = 146) and/or missing information about their hobbies (*n* = 549) were also excluded. Finally, a total of 10,405 men and 11,972 women were included in the current analysis (Figure 1).

The participants were informed of the objectives of the study, and the completion of the survey questionnaire in 1993–1994 was regarded as providing consent for participation. This study was approved by the Institutional Review Boards of the National Cancer Center, Japan, and Osaka University.

### The self-administered questionnaire survey

The participants were queried about their hobby engagement as follows: “Do you have any hobbies?”, with three possible responses: having no hobbies, having a hobby, and having many hobbies. Body mass index was calculated as the weight (kg) divided by height squared (m^2^). We calculated the total physical activity by summing all the products of daily engagement time and metabolic equivalents (METs) per hour in METs/day for each activity (strenuous exercise, sitting, standing or walking, and sleep or others).^18^ Our previous study using the 5-year follow-up questionnaire of the JPHC study, which included the same items of physical activity in this study, showed that the Spearman’s rank correlation coefficient between the total METs/day score and physical activity record was 0.46.^18^ We also asked the participants, “How many friends do you meet at least once per week?”, with three possible responses: no friends, 1–3 friends, and ≥4 friends. Type A characteristics were assessed based on four items representing the aspects of competitive drive, speed and impatience, aggressiveness, and irritability. We calculated the type A behavior pattern index by summing the scores of four items (0 to 2 for each) and divided the participants into four categories: low (score of 0 to 3), medium (score of 4), high (score of 5), and very high (score of 6 or more).^19^ The participants answered about perceived mental stress from “How much stress do you have in your daily life?”, with three responses: low, moderate, and high. We further asked for the participant, “Are you living with someone?”, with any of five possible responses: alone, spouse, child(ren), parent(s), and others. The status of hypertension, diabetes, and hypercholesterolemia were determined from the responses to the baseline questions regarding the use of medication and the medical history of the respective diseases.

### Definition of disabling dementia

In the Japanese LTCI system, when the certification of care level was applied, the preliminary care level of applicants was computed based on a structured interview survey. Subsequently, the committee of long-term care requirement certification in the local government, which is composed of experts in medical, health, and welfare areas, finalized the care level for the applicant after reviewing all the documents, including the result of the computerized preliminary care level and the statement of the primary doctor’s examination.^16^^,^^17^ We defined disabling dementia as individuals who were certified regarding their care level (excluding support levels) and ranked in IIa or worse grade of ADL in older adults with dementia by the attending physician. The first certified date of the care level meeting with our dementia criteria was used as the date of the incident disabling dementia. These criteria were previously verified in comparison with neuropsychiatrists’ diagnoses of disabling dementia, and the sensitivity and specificity were 73% and 96%, respectively.^20^

To investigate whether the association between hobby engagement and disabling dementia differed with dementia subtypes, we divided disabling dementia cases into disabling dementia without a history of stroke (possibly non-vascular type dementia) and post-stroke disabling dementia (possibly vascular-type dementia) according to the presence or absence of a history of stroke before the onset of disabling dementia. A history of stroke was obtained from 5- and 10-year follow-up questionnaires and stroke registration. The details of stroke registration have been published elsewhere.^21^

### Statistical analysis

Person-years were calculated for each individual as the duration from January 1, 2006, when the outcome data started to be available, to the date of identification of disabling dementia, the date of death, lost to follow-up, or the end of follow-up (December 31, 2016), whichever occurred first. When we used the dementia subtypes as the outcomes, the follow-up ended on December 31, 2012, instead of December 31, 2016, owing to the unavailability of the stroke registry data.

Differences in mean value or proportions of the baseline characteristics were tested using the chi-square test and the analysis of variance. The hazard ratios (HRs) and 95% confidence intervals (CIs) of incident disabling dementia were calculated according to the hobby engagement categories using the Cox proportional hazards model. The proportional hazard assumption was confirmed visually by looking at the log-log plots using LIFETEST procedure and was not rejected by assessing the weighted Schoenfeld residuals using PHREG procedures with zph-options. We tested the interaction of hobby engagement with other variables for incident disabling dementia using cross-product terms in the Cox proportional hazards model. As there was no interaction between having hobbies and sex in relation to the incident disabling dementia (*P* for interaction: 0.28 for having a hobby and 0.60 for having many hobbies), we did not conduct sex-specific analyses. The HRs and 95% CIs of the disabling dementia subtypes were calculated using a cause-specific Cox proportional hazard model. In model 1, we stratified jointly by area (four towns) and adjusted for age (years) at the questionnaire survey in 1993–1994 and sex. In model 2, we adjusted further for body mass index (<18.5, 18.5–<25.0, 25.0–<30, ≥30 kg/m^2^, and missing), smoking status (never, former, 1–19 cigarettes/day, ≥20 cigarettes/day, and missing), alcohol intake (non-drinkers, former drinker, occasional drinkers, 1–<150 g/week, 150–<300 g/week, ≥300 g/week, and missing), total physical activity (tertiles of METs/day, and missing), and histories of hypertension, diabetes, and hypercholesterolemia (yes, no, and missing, for each). In model 3, we adjusted further for living alone (yes, no, and missing), job status (yes, no, and missing), perceived mental stress (low, moderate, high, and missing), type A characteristics (low, medium, high, very high, and missing), and the number of friends (no friends, 1–3 friends, ≥4 friends, and missing). We used the category of missing data for these confounding variables in the statistical model. For sensitivity analysis, we excluded the participants with missing data. We also conducted stratified analysis by age categories at the questionnaire survey in 1993–1994 (40–64 years and 65–69 years old).

All statistical analyses were performed using the SAS (version 9.4; SAS Institute, Cary, NC, USA) in a two-sided test. The statistical significance was set at *P* < 0.05.

## RESULTS

Table 1 shows the baseline characteristics of potential confounding factors according to the hobby engagement categories. Individuals having hobbies were younger, had higher proportions of men, being physically active, current smoker, current drinker, medical history of hypercholesterolemia, being employed, living alone, having low mental stress, high type A characteristics, and having friends, and lower proportions of medical history of hypertension and medication use for hypertension compared with those having no hobbies.

**Table 1.** Characteristics of participants in 1993–1994 according to the hobby engagement categories

		Hobby categories						*P* for difference

		Having no hobbies		Having a hobby		Having many hobbies		
Number at risk		4,518		16,126		1,733		
Age, years, mean (SD)		53.1	(8.8)	52.5	(8.5)	52.7	(8.3)	<0.001
Sex, *n* (%)								
	Men	1,692	(37.5)	7,765	(48.2)	948	(54.7)	<0.001
	Women	2,826	(62.6)	8,361	(51.9)	785	(45.3)	
Body mass index								
kg/m^2^, mean (SD)		23.3	(3.1)	23.3	(2.9)	23.4	(2.9)	0.28
*n* (%)	<18.5 kg/m^2^	198	(4.4)	524	(3.3)	43	(2.5)	
	18.5–<25.0 kg/m^2^	3,098	(68.6)	11,410	(70.8)	1,235	(71.3)	
	25.0–<30.0 kg/m^2^	1,024	(22.7)	3,692	(22.9)	409	(23.6)	
	≥30.0 kg/m^2^	124	(2.7)	311	(1.9)	35	(2.0)	
	Missing data	74	(1.6)	189	(1.2)	11	(0.6)	
METs/day score								
mean (SD)		34.4	(6.6)	34.8	(6.7)	35.4	(6.7)	<0.001
*n* (%)	First tertile (range: 23.05–30.10)	1,211	(26.8)	4,412	(27.4)	441	(25.5)	
	Second tertile (range: 31.85–37.45)	1,751	(38.8)	5,861	(36.4)	592	(34.2)	
	Third tertile (range: 37.90–46.25)	1,360	(30.1)	5,334	(33.1)	651	(37.6)	
	Missing data	196	(4.3)	519	(3.2)	49	(2.8)	
Smoking status, *n* (%)								
	Never	2,950	(65.3)	9,136	(56.7)	890	(51.4)	<0.001
	Former	396	(8.8)	1,982	(12.3)	219	(12.6)	
	1–19 cigarettes/day	365	(8.1)	1,389	(8.6)	154	(8.9)	
	≥20 cigarettes/day	771	(17.1)	3,478	(21.6)	450	(26.0)	
	Missing data	36	(0.8)	141	(0.9)	20	(1.2)	
Alcohol intake, *n* (%)								
	Non-drinker	2,577	(57.0)	7,656	(47.5)	752	(43.4)	<0.001
	Former	94	(2.1)	307	(1.9)	28	(1.6)	
	Occasional	229	(5.1)	1,031	(6.4)	104	(6.0)	
	1–<150 g/week	618	(13.7)	2,924	(18.1)	338	(19.5)	
	150–<300 g/week,	444	(9.8)	1,864	(11.6)	232	(13.4)	
	≥300 g/week	344	(7.6)	1,423	(8.8)	187	(10.8)	
	Missing data	212	(4.7)	921	(5.7)	92	(5.3)	
Medical history of hypertension, *n* (%)								
	No	3,616	(80.0)	13,217	(82.0)	1,439	(83.0)	0.01
	Yes	856	(19.0)	2,785	(17.3)	278	(16.0)	
	Missing data	46	(1.0)	124	(0.8)	16	(0.9)	
Medication use for hypertension, *n* (%)								
	No	3,752	(83.1)	13,753	(85.3)	1,490	(86.0)	<0.001
	Yes	754	(16.7)	2,308	(14.3)	236	(13.6)	
	Missing data	12	(0.3)	65	(0.4)	7	(0.4)	
Medical history of diabetes, *n* (%)								
	No	4,256	(94.2)	15,211	(94.3)	1,626	(93.8)	0.49
	Yes	216	(4.8)	791	(4.9)	91	(5.3)	
	Missing data	46	(1.0)	124	(0.8)	16	(0.9)	
Medication use for diabetes, *n* (%)								
	No	4,413	(97.7)	15,782	(97.9)	1,699	(98.0)	0.41
	Yes	91	(2.0)	275	(1.7)	26	(1.5)	
	Missing data	14	(0.3)	69	(0.4)	8	(0.5)	
Medical history of hypercholesterolemia, *n* (%)								
	No	4,375	(96.8)	15,588	(96.7)	1,658	(95.7)	0.03
	Yes	97	(2.2)	414	(2.6)	59	(3.4)	
	Missing data	46	(1.0)	124	(0.8)	16	(0.9)	
Medication use for hypercholesterolemia, *n* (%)								
	No	4,418	(97.8)	15,704	(97.4)	1,675	(96.7)	0.14
	Yes	86	(1.9)	354	(2.2)	50	(2.9)	
	Missing data	14	(0.3)	68	(0.4)	8	(0.5)	
Job status, *n* (%)								
	Unemployed	999	(22.1)	3,296	(20.4)	329	(19.0)	0.009
	Employed	3,471	(76.8)	12,692	(78.7)	1,394	(80.4)	
	Missing data	48	(1.1)	138	(0.9)	10	(0.6)	
Living alone, *n* (%)								
	No	4,385	(97.1)	15,625	(96.9)	1,656	(95.6)	<0.001
	Yes	116	(2.6)	483	(3.0)	75	(4.3)	
	Missing data	17	(0.4)	18	(0.1)	2	(0.1)	
Perceived mental stress, *n* (%)								
	Low	692	(15.3)	2,598	(16.1)	357	(20.6)	<0.001
	Moderate	2,800	(62.0)	10,415	(64.6)	923	(53.3)	
	High	1,007	(22.3)	3,044	(18.9)	445	(25.7)	
	Missing data	19	(0.4)	69	(0.4)	8	(0.5)	
Type A characteristics, *n* (%)								
	Low	1,085	(24.0)	3,254	(20.2)	349	(20.1)	<0.001
	Moderate	1,743	(38.6)	6,543	(40.6)	519	(30.0)	
	High	647	(14.3)	2,624	(16.3)	290	(16.7)	
	Very high	752	(16.6)	3,054	(18.9)	504	(29.1)	
	Missing data	291	(6.4)	651	(4.0)	71	(4.1)	
Number of friends, *n* (%)								
	No friends	1,245	(27.6)	1,913	(11.9)	122	(7.0)	<0.001
	1–3 friends	2,446	(54.1)	9,035	(56.0)	648	(37.4)	
	≥4 friends	744	(16.5)	4,992	(31.0)	947	(54.7)	
	Missing data	83	(1.8)	186	(1.2)	16	(0.9)	

METs, metabolic equivalents; SD, standard deviation.

During the median 11.0 years of follow-up starting 12 years after exposure assessment, we confirmed 3,095 cases of disabling dementia. Having hobbies was inversely associated with the risk of incident disabling dementia (Table 2). Compared with “having no hobbies”, multivariable-adjusted HRs of incident disabling dementia were 0.81 (95% CI, 0.75–0.88) for “having a hobby” and 0.77 (95% CI, 0.66–0.89) for “having many hobbies” after adjustment for age, sex, lifestyle factors, and medical history. A further adjustment for psychosocial factors did not substantially changes the association. In the stratified analysis by age, the inverse association was not significantly different between the participants aged 40–64 years and those 65–69 years at the questionnaire survey (*P* for interaction: 0.32 for having a hobby and 0.42 for having many hobbies).

**Table 2.** Hazard ratios (HRs) and 95% confidence intervals (CIs) for incidence disabling dementia according to hobby engagement categories among Japanese aged 40–69 years

		Hobby categories		

		Having no hobbies	Having a hobby	Having many hobbies
Total				
	Person-years	41,483	153,232	16,340
	Number at risk	4,518	16,126	1,733
	Number of cases	784	2,099	212
	^a^Model 1 HR (95% CI)	reference	0.79 (0.73–0.86)	0.75 (0.65–0.88)
	^b^Model 2 HR (95% CI)	reference	0.81 (0.75–0.88)	0.77 (0.66–0.89)
	^c^Model 3 HR (95% CI)	reference	0.82 (0.75–0.89)	0.78 (0.67–0.91)
40–64 years in 1993–1994				
	Person-years	37,636	141,642	15,113
	Number at risk	3,898	14,344	1,553
	Number of cases	451	1,294	140
	^a^Model 1 HR (95% CI)	reference	0.77 (0.69–0.85)	0.78 (0.65–0.94)
	^b^Model 2 HR (95% CI)	reference	0.79 (0.70–0.88)	0.82 (0.68–0.99)
	^c^Model 3 HR (95% CI)	reference	0.79 (0.71–0.88)	0.83 (0.68–1.01)
			└0.79 (0.71–0.89)┘	
65–69 years in 1993–1994				
	Person-years	3,847	11,590	1,226
	Number at risk	620	1,782	180
	Number of cases	333	805	72
	^a^Model 1 HR (95% CI)	reference	0.83 (0.73–0.94)	0.70 (0.54–0.90)
	^b^Model 2 HR (95% CI)	reference	0.83 (0.73–0.95)	0.68 (0.53–0.89)
	^c^Model 3 HR (95% CI)	reference	0.84 (0.73–0.96)	0.68 (0.52–0.90)
			└0.82 (0.72–0.94)┘	

^a^Adjusted for age in 1993–1994 and sex.

^b^Adjusted further for body mass index, smoking status, alcohol intake, total physical activity, history of hypertension, diabetes, and hypercholesterolemia.

^c^Adjusted further for living alone, job status, perceived mental stress, type A characteristics, and number of friends.

Regarding disabling dementia subtypes, an inverse association of having hobbies was observed for disabling dementia without a history of stroke, but not for post-stroke disabling dementia (Table 3). Model 3 HRs of incident disabling dementia without a history of stroke were 0.77 (95% CI, 0.68–0.88) for “having a hobby” and 0.77 (95% CI, 0.60–0.98) for “having many hobbies”. After stratification by age at the questionnaire survey, the inverse association for disabling dementia without a history of stroke was not significantly different between age categories. For having hobbies combining two hobby engagement categories compared to “having no hobbies” group, multivariable-adjusted HRs of disabling dementia without a history of stroke were 0.71 (95% CI, 0.60–0.85) for participants aged 40–64 years and 0.82 (95% CI, 0.68–0.99) for those aged 65–69 years (*P* for interaction = 0.09).

**Table 3.** Hazard ratios (HRs) and 95% confidence intervals (CIs) for incidence disabling dementia subtypes according to hobby engagement categories among Japanese aged 40–69 years

			Hobby categories		

			Having no hobbies	Having a hobby	Having many hobbies
Disabling dementia without a history of stroke					
	Total				
		Person-years	28,317	103,176	11,017
		Number at risk	4,518	16,126	1,733
		Number of cases	365	878	90
		^a^Model 1 HR (95% CI)	reference	0.75 (0.66–0.84)	0.74 (0.59–0.93)
		^b^Model 2 HR (95% CI)	reference	0.76 (0.67–0.86)	0.76 (0.60–0.95)
		^c^Model 3 HR (95% CI)	reference	0.77 (0.68–0.88)	0.77 (0.60–0.98)
				└0.77 (0.68–0.88)┘	
	40–64 years in 1993–1994				
		Person-years	25,270	94,140	10,101
		Number at risk	3,898	14,344	1,553
		Number of cases	196	476	53
		^a^Model 1 HR (95% CI)	reference	0.67 (0.57–0.80)	0.70 (0.51–0.95)
		^b^Model 2 HR (95% CI)	reference	0.69 (0.59–0.82)	0.75 (0.55–1.01)
		^c^Model 3 HR (95% CI)	reference	0.71 (0.60–0.84)	0.75 (0.55–1.04)
				└0.71 (0.60–0.85)┘	
	65–69 years in 1993–1994				
		Person-years	3,047	9,036	916
		Number at risk	620	1,782	180
		Number of cases	169	402	37
		^a^Model 1 HR (95% CI)	reference	0.84 (0.70–1.00)	0.78 (0.55–1.12)
		^b^Model 2 HR (95% CI)	reference	0.84 (0.70–1.01)	0.75 (0.52–1.08)
		^c^Model 3 HR (95% CI)	reference	0.83 (0.69–0.998)	0.75 (0.52–1.10)
				└0.82 (0.68–0.99)┘	
Post-stroke disabling dementia				
	Total				
		Number of cases	114	318	34
		^a^Model 1 HR (95% CI)	reference	0.83 (0.67–1.02)	0.84 (0.57–1.23)
		^b^Model 2 HR (95% CI)	reference	0.87 (0.70–1.08)	0.87 (0.59–1.28)
		^c^Model 3 HR (95% CI)	reference	0.89 (0.71–1.11)	0.93 (0.62–1.38)
				└0.89 (0.71–1.11)┘	
	40–64 years in 1993–1994				
		Number of cases	60	191	18
		^a^Model 1 HR (95% CI)	reference	0.83 (0.62–1.12)	0.70 (0.42–1.19)
		^b^Model 2 HR (95% CI)	reference	0.88 (0.65–1.18)	0.74 (0.44–1.26)
		^c^Model 3 HR (95% CI)	reference	0.89 (0.66–1.20)	0.76 (0.44–1.32)
				└0.88 (0.65–1.18)┘	
	65–69 years in 1993–1994				
		Number of cases	54	127	16
		^a^Model 1 HR (95% CI)	reference	0.81 (0.59–1.12)	1.06 (0.60–1.85)
		^b^Model 2 HR (95% CI)	reference	0.84 (0.60–1.16)	1.01 (0.57–1.78)
		^c^Model 3 HR (95% CI)	reference	0.85 (0.61–1.19)	1.09 (0.60–1.98)
				└0.87 (0.62–1.21)┘	

^a^Adjusted for age in 1993–1994 and sex.

^b^Adjusted further for body mass index, smoking status, alcohol intake, total physical activity, history of hypertension, diabetes, and hypercholesterolemia.

^c^Adjusted further for living alone, job status, perceived mental stress, type A characteristics, and number of friends.

After excluding participants with missing data of confounding variables, the results did not change materially (eTable 1 and eTable 2).

## DISCUSSION

In the current study, hobby engagement in the age range of 40–69 years (mean, 53 years) was associated with a lower risk of disabling dementia among Japanese men and women during 12 to 23 years after hobby assessment. Further, the inverse association was apparent for disabling dementia without a history of stroke, but not for post-stroke disabling dementia. The association between hobby engagement and disabling dementia were similar between middle (40–64 years) and older ages (65–69 years) at the questionnaire survey.

In the Monongahela Valley Independent Elders Survey project of 942 individuals in the United States aged ≥65 years with an average of 6 years of follow-up, a longer time commitment to hobbies was associated with a lower risk of dementia.^8^ A 6-year follow-up for Japanese men and women aged ≥65 years in the Japan Gerontological Evaluation Study (JAGES) showed that several types of hobbies, such as ground golf and travel, were associated with a lower risk of dementia.^6^ Moreover, the UK Million Women Study showed that the participation in groups for art, craft, or music at a mean age of 60 years and reading at a mean age of 64 years were associated with a lower risk of dementia in 0–4 and 5–9 years follow-up, but not in ≥10 years follow-up.^22^ The Betula prospective cohort study of individuals aged ≥65 years also reported that leisure activity was associated with a lower risk of dementia in the first period (1–5 years after baseline), but not in the second and third periods (6–10 and 11–15 year after baseline).^5^ The findings from the above two studies were different from ours probably because of different timing for exposure determination in late life and mid-life. Our finding in mid-life corroborated the result from the Gothenburg H70 Birth Cohort Study of 800 Swedish women aged 38–54 years, which reported that cognitive and physical activity in mid-life were associated with a lower risk of dementia in a mean of 44 years of follow-up.^13^

In the present study, we found that hobby engagement was inversely associated with the risk of disabling dementia without a history of stroke, which may correspond to non-vascular type dementia, most likely Alzheimer’s disease. In most previous studies, cognitive activity was associated with a lower risk of Alzheimer’s disease,^2^^–^^4^^,^^7^^,^^13^ while only one study reported a protective effect of cognitive activity on vascular dementia and mixed dementia.^3^ On the other hand, physical activity was inversely associated with a risk of Alzheimer’s disease,^2^^,^^10^ vascular dementia,^12^ dementia with cerebrovascular disease,^13^ and mixed dementia.^13^

Several possible mechanisms can be addressed for the protective effect of hobby engagement on dementia. First, hobby engagement, as an enjoyable leisure activity at age of 19–89 years, was correlated with enhanced life engagement (purpose in life),^1^ which was associated with a lower risk of dementia^14^ partly due to lower levels of inflammatory markers.^23^ Furthermore, high average cognitive activity across ages of 6, 12, 18, and 40 years was associated with lower β-amyloid accumulation measured using positron emission tomography among people aged 50 years or older,^24^ suggesting that cognitive activity from the early life makes cognitive reserve and prevents dementia. On the other hand, high physical activity (such as ≥2.5 hours of moderate to vigorous physical activity/week and participation in leisure time physical activity at ≥2 times/week) in mid-life was associated with lower levels of inflammatory markers^25^ and a larger volume of gray matter^26^ in late life compared to the lower physical activity. Further, physical activity in old age of 60–95 years was associated with lower β-amyloid accumulation.^27^ Physical activity decreases the risk of the development of hypertension, diabetes, and obesity,^28^^–^^30^ all of which were risk factors for the development of dementia.^31^

The strengths of this study are the large sample size, minimizing the probability of chance findings. Incident disabling dementia was identified using routinely collected government data with validated methods, which reduced the possibility of reporting error of outcome.

This study has several limitations. First, we had no information about the cognitive function and a history of dementia at the time of the questionnaire survey in 1993–1994. Cognitive decline and dementia may lead to reduced hobby engagement; therefore, reverse causation cannot be inevitable. In the present study, however, dementia outcome was assessed 12 years after the exposure assessment, and the impact of reverse causation may be small. Second, we could not discuss the specific associations of the actual number, types, frequency, and intensity of hobbies with the risk of disabling dementia because we did not ask for these information. Previous studies showed that the number of hobbies,^6^ time commitment to hobbies,^8^ and the intensity of cognitive and physical activity^13^ were associated with a lower risk of dementia. Third, we assessed the hobby only at the questionnaire survey in 1993–1994, so we could not take into account the changes in hobby engagement during the follow-up. Some of the participants may quit or start hobbies during the follow-up. For example, starting a new hobby in late life could lead to lowering the dementia risk, attenuating the association between hobby engagement and the dementia risk. Fourth, we did not have a precise diagnosis of dementia subtypes, including Alzheimer’s dementia, vascular dementia, and mixed dementia, although we found an inverse association between hobby engagement and the risk of disabling dementia without a history of stroke, a surrogate outcome for Alzheimer’s disease. Finally, residual and unmeasured confounding factors may exist, such as education levels, the presence of psychiatric disorders, and use of psychiatric medications.

In conclusion, hobby engagement in both mid-life and late life was associated with a lower risk of disabling dementia without a history of stroke among the Japanese population.

## SUPPLEMENTARY MATERIAL

eTable 1. Hazard ratios (HRs) and 95% confidence intervals (CIs) for incidence disabling dementia according to hobby engagement categories among Japanese aged 40–69 years excluding individuals with missing data of confounding variables

eTable 2. Hazard ratios (HRs) and 95% confidence intervals (CIs) for incidence disabling dementia subtypes according to hobby engagement categories among Japanese aged 40–69 years excluding individuals with missing data of confounding variables
