# Supplementary material for: Hobby Engagement and Risk of Disabling Dementia
Source: J Epidemiol. 2023 Sep 5;33(9):456–63. doi: 10.2188/jea.JE20210489 (PMC10409527; doi:10.2188/jea.JE20210489)
Supplement: Supplementary file 1 [file je-33-456-s001.pdf]

eTable 1. Hazard ratios (HRs) and 95% confidence intervals (CIs) for incidence disabling dementia according to hobby engagement categories among Japanese aged 40–69 years excluding individuals with missing data of confounding variables

	Hobby categories		
	Having no hobbies	Having a hobby	Having many hobbies
Total			
Person-years	34,100	130,175	14,088
Number at risk	3,667	13,584	1,481
Number of cases	592	1,677	172
[a]Model 1 HR (95% CI)	reference	0.79 (0.72 – 0.87)	0.73 (0.62 – 0.87)
[b]Model 2 HR (95% CI)	reference	0.81 (0.73 – 0.89)	0.74 (0.63 – 0.88)
[c]Model 3 HR (95% CI)	reference	0.81 (0.73 – 0.89)	0.75 (0.63 – 0.90)
40–64 years in 1993–1994			
Person-years	31,208	120,690	13,009
Number at risk	3,210	12,158	1,331
Number of cases	350	1,045	115
[a]Model 1 HR (95% CI)	reference	0.77 (0.68 – 0.87)	0.78 (0.63 – 0.97)
[b]Model 2 HR (95% CI)	reference	0.78 (0.69 – 0.88)	0.82 (0.66 – 1.01)
[c]Model 3 HR (95% CI)	reference	0.79 (0.70 – 0.90)	0.84 (0.67 – 1.04)
		└ 0.79 (0.70 – 0.90) ┘	
65–69 years in 1993–1994			
Person-years	2,892	9,485	1,079
Number at risk	457	1,426	150
Number of cases	242	632	57
[a]Model 1 HR (95% CI)	reference	0.83 (0.71 – 0.96)	0.66 (0.49 – 0.88)
[b]Model 2 HR (95% CI)	reference	0.83 (0.71 – 0.97)	0.64 (0.48 – 0.86)
[c]Model 3 HR (95% CI)	reference	0.83 (0.71 – 0.97)	0.64 (0.47 – 0.86)
		└ 0.81 (0.70 – 0.95) ┘	

[a] Adjusted for age in 1993–1994 and sex.

[b] Adjusted further for body mass index, smoking status, alcohol intake, total physical activity, history of hypertension, diabetes, and hypercholesterolemia.

[c] Adjusted further for living alone, job status, perceived mental stress, type A characteristics, and number of friends.

eTable 2. Hazard ratios (HRs) and 95% confidence intervals (CIs) for incidence disabling dementia subtypes according to hobby engagement categories among Japanese aged 40–69 years excluding individuals with missing data of confounding variables

		Hobby categories		
		Having no hobbies	Having a hobby	Having many hobbies
Disabling dementia without a history of stroke				
Total				
	Person-years	23,172	87,408	9,487
	Number at risk	3,667	13,584	1,481
	Number of cases	280	682	75
	[a]Model 1 HR (95% CI)	reference	0.71 (0.62 – 0.82)	0.72 (0.56 – 0.93)
	[b]Model 2 HR (95% CI）	reference	0.72 (0.62 – 0.82)	0.73 (0.56 – 0.94)
	[c]Model 3 HR (95% CI)	reference	0.72 (0.62 – 0.83)	0.73 (0.56 – 0.96)
			└ 0.72 (0.62 – 0.83) ┘	
40–64 years in 1993–1994				
	Person-years	20,899	80,075	8,681
	Number at risk	3,210	12,158	1,331
	Number of cases	153	368	45
	[a]Model 1 HR (95% CI)	reference	0.64 (0.53 – 0.78)	0.72 (0.52 – 1.00)
	[b]Model 2 HR (95% CI）	reference	0.65 (0.54 – 0.78)	0.75 (0.53 – 1.04)
	[c]Model 3 HR (95% CI)	reference	0.66 (0.54 – 0.80)	0.75 (0.53 – 1.06)
			└ 0.66 (0.55 – 0.81) ┘	
65–69 years in 1993–1994				
	Person-years	2,273	7,333	806
	Number at risk	457	1,426	150
	Number of cases	127	314	30
	[a]Model 1 HR (95% CI)	reference	0.80 (0.65 – 0.99)	0.72 (0.48 – 1.07)
	[b]Model 2 HR (95% CI）	reference	0.81 (0.65 – 0.99)	0.70 (0.47 – 1.05)
	[c]Model 3 HR (95% CI)	reference	0.80 (0.64 – 0.99)	0.71 (0.47 – 1.09)
			└ 0.79 (0.64 – 0.98) ┘	

Post-stroke disabling dementia			
Total			
Number of cases	78	249	26
[a]Model 1 HR (95% CI)	reference	0.89 (0.69 – 1.15)	0.85 (0.54 – 1.32)
[b]Model 2 HR (95% CI)	reference	0.94 (0.73 – 1.22)	0.87 (0.55 – 1.35)
[c]Model 3 HR (95% CI)	reference	0.94 (0.73 – 1.23)	0.90 (0.57 – 1.42)
		└ 0.94 (0.72 – 1.22) ┘	
40–64 years in 1993–1994			
Number of cases	40	153	15
[a]Model 1 HR (95% CI)	reference	0.96 (0.68 – 1.36)	0.83 (0.46 – 1.51)
[b]Model 2 HR (95% CI)	reference	1.02 (0.72 – 1.44)	0.88 (0.49 – 1.61)
[c]Model 3 HR (95% CI)	reference	1.02 (0.71 – 1.47)	0.91 (0.49 – 1.68)
		└ 1.01 (0.71 – 1.45) ┘	
65–69 years in 1993–1994			
Number of cases	38	96	11
[a]Model 1 HR (95% CI)	reference	0.80 (0.55 – 1.17)	0.86 (0.44 – 1.68)
[b]Model 2 HR (95% CI)	reference	0.85 (0.58 – 1.25)	0.82 (0.42 – 1.63)
[c]Model 3 HR (95% CI)	reference	0.85 (0.57 – 1.25)	0.83 (0.41 – 1.68)
		└ 0.85 (0.57 – 1.25) ┘	

[a] Adjusted for age in 1993–1994 and sex.

[b] Adjusted further for body mass index, smoking status, alcohol intake, total physical activity, history of hypertension, diabetes, and hypercholesterolemia.

[c] Adjusted further for living alone, job status, perceived mental stress, type A characteristics, and number of friends.
